# Approximate representations of shaped pulses using the homotopy analysis method

**DOI:** 10.5194/mr-2-175-2021

**Published:** 2021-04-16

**Authors:** Timothy Crawley, Arthur G. Palmer III

**Affiliations:** Department of Biochemistry and Molecular Biophysics, Columbia University, 630 West 168th Street, New York, NY 10032, United States

## Abstract

The evolution of nuclear spin magnetization during a radiofrequency pulse in the absence of relaxation or coupling interactions can be described by three Euler angles. The Euler angles, in turn, can be obtained from the solution of a Riccati differential equation; however, analytic solutions exist only for rectangular and hyperbolic-secant pulses. The homotopy analysis method is used to obtain new approximate solutions to the Riccati equation for shaped radiofrequency pulses in nuclear magnetic resonance (NMR) spectroscopy. The results of even relatively low orders of approximation are highly accurate and can be calculated very efficiently. The results are extended in a second application of the homotopy analysis method to represent relaxation as a perturbation of the magnetization trajectory calculated in the absence of relaxation. The homotopy analysis method is powerful and flexible and is likely to have other applications in magnetic resonance.

## 1 Introduction

Numerous aspects of nuclear magnetic resonance (NMR) spectroscopy are formulated in terms of differential equations, few of which have closed-form analytical solutions. In an era characterized by ever-increasing computational capabilities, numerical solutions to such differential equations are always possible and are frequently the preferred approach for applications such as data analysis. However, approximate solutions can provide useful formulas and insights that are difficult to discern from purely numerical results.

As one example, the net evolution of the magnetization of an isolated spin during a radiofrequency pulse, i.e. in the absence of relaxation and scalar or other coupling interactions, can be described by three rotations with Euler angles $\alpha (\tau_{p})$ , $\beta (\tau_{p})$ , and $\gamma (\tau_{p})$ , in which $\tau_{p}$ is the pulse length [Bibr bib1.bibx21]. Shaped pulses, in which the amplitude (Rabi frequency), phase, or radiofrequency are time dependent, are widely applied in modern NMR spectroscopy and other magnetic resonance techniques [Bibr bib1.bibx5]. The Euler angles for an arbitrary shaped pulse can be extracted from a numerical calculation in which the shaped pulse is represented by a series of K short rectangular pulses with appropriate amplitudes and phases. Thus, the propagator for a shaped pulse expressed in the Cartesian basis is given by the following [Bibr bib1.bibx17]:

1 $$\begin{array}{rl} U & =e^{-i\gamma (\tau_{p})I_{z}}e^{-i\beta (\tau_{p})I_{x}}e^{-i\alpha (\tau_{p})I_{z}}\\ & =\left[\begin{array}{rr} e^{-i\chi^{+}(\tau_{p})}\mathrm{cos}(\frac{\beta (\tau_{p})}{2}) & -ie^{i\chi^{-}(\tau_{p})}\mathrm{sin}(\frac{\beta (\tau_{p})}{2})\\ -ie^{-i\chi^{-}(\tau_{p})}\mathrm{sin}(\frac{\beta (\tau_{p})}{2}) & e^{i\chi^{+}(\tau_{p})}\mathrm{cos}(\frac{\beta (\tau_{p})}{2}) \end{array}\right]\\ & =\prod_{k=1}^{K}U_{k}, \end{array}$$

in which $\chi^{\pm }(\tau_{p})$ is given as follows:

2 $$\chi^{\pm }(\tau_{p})=[\alpha (\tau_{p})\pm \gamma (\tau_{p})]/2,$$

$I_{k}$ are the Cartesian spin operators, and the product is time-ordered from right to left. The propagator for the k th rectangular pulse segment is given as follows:

3 $$U_{k}=\left[\begin{array}{rr} \kappa^{*}(\tau_{p}) & -i\eta^{*}(\tau_{p})\\ -i\eta (\tau_{p}) & \kappa (\tau_{p}) \end{array}\right],$$

in which $\kappa (\tau_{p})$ and $\eta (\tau_{p})$ are given by the following:

4 $$\begin{array}{rl} & \kappa (\tau_{p})=\mathrm{cos}(\omega_{ek}\Delta t_{k}/2)+i\mathrm{cos}\theta_{k}\mathrm{sin}(\omega_{ek}\Delta t_{k}/2)\\ & \eta (\tau_{p})=e^{i\phi_{k}}\mathrm{sin}\theta_{k}\mathrm{sin}(\omega_{ek}\Delta t_{k}/2). \end{array}$$

In Eq. (4), $\omega_{1k}$ , $\phi_{k}$ , and $\Delta t_{k}$ are the radiofrequency field strength, phase angle, and duration of the k th pulse segment; $\Omega_{k}$ is the resonance offset in the rotating frame of reference during the k th pulse segment (and is constant if the offset is fixed); $\omega_{ek}=(\omega_{1k}^{2}+\Omega_{k}^{2}{)}^{1/2}$ is the effective field; and $\theta_{k}={\mathrm{tan}}^{-1}(\omega_{1k}/\Omega_{k})$ is the tilt angle. Values of $\alpha (\tau_{p})$ , $\beta (\tau_{p})$ , and $\gamma (\tau_{p})$ are then obtained from the matrix elements of U .

Alternatively, the Euler angles can be determined from the solution of the following Riccati equation [Bibr bib1.bibx21]:

5 $$\frac{df(t)}{dt}=\frac{1}{2}\omega^{+}(t)f^{2}(t)+i\Omega (t)f(t)+\frac{1}{2}\omega^{-}(t),$$

in which the following applies:

6 $$f(t)=\mathrm{tan}\left(\frac{\beta (t)}{2}\right)e^{i\gamma (t)},$$

where $\omega^{\pm }(t)=\omega_{x}(t)\pm i\omega_{y}(t)$ and $\omega_{x}(t)$ and $\omega_{y}(t)$ are the Cartesian amplitude components of the radiofrequency field in the rotating frame of reference. After solution of the Riccati equation, $\beta (\tau_{p})$ and $\gamma (\tau_{p})$ are obtained from the magnitude and argument of $f(\tau_{p})$ , and the value of $\alpha (\tau_{p})$ is obtained by integration as follows:

7 $$\alpha (\tau_{p})=\int_{0}^{\tau_{p}}dt\{\omega_{x}(t)\mathrm{sin}[\gamma (t)]-\omega_{y}(t)\mathrm{cos}[\gamma (t)]\}/\mathrm{sin}[\beta (t)].$$

The Riccati equation can be transformed into a second-order differential equation as follows:

8 $$\frac{d^{2}y(t)}{dt^{2}}-\left[\frac{d\mathrm{ln}\left[\omega^{-}(t)\right]}{dt}+i\Omega (t)\right]\frac{dy(t)}{dt}+\frac{1}{4}|\omega (t){|}^{2}y(t)=0,$$

by using the following definition:

9 $$\frac{d\mathrm{ln}\left[y(t)\right]}{dt}=-\frac{1}{2}\omega^{-}(t)f(t).$$

A more compact form is obtained by defining the following:

10 $${\hat{\omega }}^{-}(t)=\mathrm{exp}\left[i\int_{0}^{t}\Omega (t^{\prime })dt^{\prime }\right]\omega^{-}(t),$$

to yield the following:

11 $$\frac{d^{2}y(t)}{dt^{2}}-\frac{d\mathrm{ln}\left[{\hat{\omega }}^{-}(t)\right]}{dt}\frac{dy(t)}{dt}+\frac{1}{4}|\hat{\omega }(t){|}^{2}y(t)=0.$$

The Riccati differential equation only can be solved analytically for a single rectangular or hyperbolic-secant pulse [Bibr bib1.bibx16]. Approximate solutions for arbitrary shaped pulses have been derived by the perturbation theory for the limits of small, using Eq. ([Disp-formula Ch1.E11]), and large, using Eq. ([Disp-formula Ch1.E5]), resonance offsets [Bibr bib1.bibx11]; however, perturbation theory is unwieldy to apply to a high order and, obviously, depends on the perturbation parameters being small in some respects.

The homotopy analysis method (HAM) is a fairly recent development, first reported in 1992 [Bibr bib1.bibx12], for approximating solutions to differential equations, particularly nonlinear ones. HAM does not depend on small parameters, unlike perturbation theory, and has proven powerful in a number of applications outside of NMR spectroscopy [Bibr bib1.bibx13]. The present paper illustrates HAM by application to the solutions of Eqs. ([Disp-formula Ch1.E5]) and ([Disp-formula Ch1.E11]) and, subsequently, by extension to the Bloch equations, including relaxation.

## 2 Theory

In topology, a pair of functions defining different topological spaces are said to be homotopic if the shape defined by one function can be continuously transformed (deformed in the lexicon of topology) into the shape defined by the other. Analogously, the essence of HAM is to map a function of interest, here y(t) , to a second function, Φ(t;q) , which has a known solution and is a function of both t and an embedding parameter q∈[0,1] .

This relationship is established by constructing the homotopy as follows [Bibr bib1.bibx13]:

12 $$\begin{array}{rl} H\left[\Phi (t;q):q\right]=(1-q) & L\left[\Phi (t;q)-y_{0}(t)\right]\\ & \;-qc_{0}H(t)N\left[\Phi (t;q)\right], \end{array}$$

in which L[] is a linear (differential) operator, and N[] is an (nonlinear differential) operator satisfying the following:

13L0=014Ny(t)=0,

where $y_{0}(t)$ is an initial approximation for the desired solution y(t) , $c_{0}\neq 0$ is a convergence control parameter, and H(t)≠0 is an auxiliary function (vide infra). When q=0 , the homotopy becomes as follows:

15 $$H\left[\Phi (t;0):0\right]=L\left[\Phi (t;0)-y_{0}(t)\right].$$

Therefore, when $H\left[\Phi (t;0):0\right]=0$ , Eq. (13) requires $\Phi (t,0)=y_{0}(t)$ . Similarly, when q=1 , the homotopy becomes as follows:

16 $$H\left[\Phi (t;1):1\right]=-c_{0}H(t)N\left[\Phi (t;1)\right].$$

Therefore, when $H\left[\Phi (t;1):1\right]=0$ , Eq. (14) requires Φ(t,1)=y(t) . Stated more succinctly, as q increases from 0→1 , Φ(t;q) deforms from the initial approximation $y_{0}(t)$ to the exact solution y(t) . To proceed, the Maclaurin series for Φ(t;q) is assumed to exist as follows:

17 $$\Phi (t;q)=\sum_{n=0}^{\infty }y_{n}(t)q^{n},$$

in which $y_{n}(t)$ is given by the following:

18 $$y_{n}(t)=\frac{1}{n!}\frac{d^{n}\Phi (t;q)}{dq^{n}}{|}_{q=0}.$$

Conditions concerning convergence of the series are discussed by [Bibr bib1.bibx13]. Equation ([Disp-formula Ch1.E17]) has the desired property $\Phi (t;0)=y_{0}(t)$ and yields the following:

19 $$\Phi (t;1)=y(t)=\sum_{n=0}^{\infty }y_{n}(t).$$

HAM then consists of successively determining $y_{n}(t)$ , beginning with the initial approximation $y_{0}(t)$ , until y(t) is approximated to the desired accuracy. The choices of L[] , $y_{0}(t)$ , $c_{0}$ , and H(t) provide considerable flexibility in finding approximate solutions to differential equations. For simplicity in the following, the auxiliary function is H(t)=1 .

The iterative algorithm in HAM is illustrated by application to the second-order differential form of the Riccati equation. In the first example, the nonlinear operator is obtained from Eq. ([Disp-formula Ch1.E11]) as follows:

20 $$N\left[g(t)\right]=\frac{d^{2}g(t)}{dt^{2}}-\frac{d\mathrm{ln}\left[{\hat{\omega }}^{-}(t)\right]}{dt}\frac{dg(t)}{dt}+\frac{1}{4}|\hat{\omega }(t){|}^{2},$$

in which g(t) is an arbitrary function. The linear operator is chosen to be the following:

21 $$L\left[g(t)\right]=\frac{d^{2}g(t)}{dt^{2}}-\frac{d\mathrm{ln}\left[{\hat{\omega }}^{-}(t)\right]}{dt}\frac{dg(t)}{dt},$$

and the initial approximation is $y_{0}(t)=1$ .

From the relationships of Eqs. (13) and (14) embedded in the initial homotopy, Eq. (12), the zeroth-order deformation equation is defined as follows [Bibr bib1.bibx13]:

22 $$(1-q)L\left[\Phi (t;q)-y_{0}(t)\right]=qc_{0}N\left[\Phi (t;q)\right].$$

The derivative of Eq. ([Disp-formula Ch1.E22]), with respect to q , yields the first-order deformation equation as follows:

23 $$\begin{array}{rl} & -L\left[\Phi (t;q)-y_{0}(t)\right]+(1-q)L\left[\frac{d\Phi (t;q)}{dq}\right]\\ & \;\;\;\;\;=c_{0}N\left[\Phi (t;q)\right]+qc_{0}\frac{d}{dq}N\left[\Phi (t;q)\right]. \end{array}$$

The limit q→0 gives the following:

-LΦ(t;0)-y0(t)+LdΦ(t;q)dq|q=0=c0NΦ(t;0)24Ly1(t)=c0Ny0(t),

in which the second line is obtained using $\Phi (t;0)=y_{0}(t)$ and Eq. ([Disp-formula Ch1.E18]). Substituting for N[] , L[] , and $y_{0}(t)$ yields the following:

25 $$\begin{array}{rl} & \frac{d^{2}y_{1}(t)}{dt^{2}}-\frac{d\mathrm{ln}\left[{\hat{\omega }}^{-}(t)\right]}{dt}\frac{dy_{1}(t)}{dt}\\ & \;\;\;\;\;=c_{0}\left(\frac{d^{2}y_{0}(t)}{dt^{2}}-\frac{d\mathrm{ln}\left[{\hat{\omega }}^{-}(t)\right]}{dt}\frac{dy_{0}(t)}{dt}+\frac{1}{4}|\hat{\omega }(t){|}^{2}y_{0}(t)\right)\\ & \;\;\;\;\;=\frac{c_{0}}{4}|\hat{\omega }(t){|}^{2}, \end{array}$$

in which the final line is obtained using $dy_{0}(t)/dt=0$ . This differential equation does not contain a term proportional to $y_{1}(t)$ . Hence, the homogenous equation (setting the right-hand side to 0) can be solved by two successive integrations, and the inhomogeneous solution is obtained by the technique of variation of parameters [Bibr bib1.bibx1]. The solution is as follows:

26 $$y_{1}(t)=\frac{c_{0}}{4}\int_{0}^{t}{\hat{\omega }}^{-}(t^{\prime })\int_{0}^{t^{\prime }}{\hat{\omega }}^{+}(t^{\prime \prime })dt^{\prime \prime }dt^{\prime }.$$

The higher-order approximations $y_{n}(t)$ are obtained in a similar fashion. The n th derivative, with respect to q of Eq. ([Disp-formula Ch1.E22]), yields the following (for n>1 ):

27 $$\begin{array}{rl} & -nL\left[\frac{d^{n-1}\Phi (t;q)}{dq^{n-1}}\right]+(1-q)L\left[\frac{d^{n}\Phi (t;q)}{dq^{n}}\right]\\ & \;\;\;\;\;\;=nc_{0}\frac{d^{n-1}}{dq^{n-1}}N\left[\Phi (t;q)\right]+qc_{0}\frac{d^{n}}{dq^{n}}N\left[\Phi (t;q)\right]. \end{array}$$

Executing the derivatives, taking the limit q→0 , and dividing both sides of the equation by n! gives the following:

28 $$\begin{array}{rl} \frac{d^{2}y_{n}(t)}{dt^{2}} & -\frac{d\mathrm{ln}\left[{\hat{\omega }}^{-}(t)\right]}{dt}\frac{dy_{n}(t)}{dt}\\ & =(c_{0}+1)\{\frac{d^{2}y_{n-1}(t)}{dt^{2}}-\frac{d\mathrm{ln}\left[{\hat{\omega }}^{-}(t)\right]}{dt}\frac{dy_{n-1}(t)}{dt}\}\\ & \;\;\;\;+\frac{1}{4}c_{0}|\hat{\omega }(t){|}^{2}y_{n-1}(t), \end{array}$$

with the solution obtained by the same approach as for Eq. ([Disp-formula Ch1.E26]), as follows:

29 $$\begin{array}{rl} y_{n}(t)=(c_{0}+ & 1)y_{n-1}(t)\\ & +\frac{c_{0}}{4}\int_{0}^{t}{\hat{\omega }}^{-}(t^{\prime })\int_{0}^{t^{\prime }}{\hat{\omega }}^{+}(t^{\prime \prime })y_{n-1}(t^{\prime \prime })dt^{\prime \prime }dt^{\prime }. \end{array}$$

Successive use of Eqs. ([Disp-formula Ch1.E26]) and ([Disp-formula Ch1.E29]) allows y(t) and, hence, f(t) to be determined to arbitrary accuracy, as follows:

30 $$f(t)=\left(\frac{-2}{\omega^{-}(t)}\right)\frac{d\mathrm{ln}\left[y(t)\right]}{dt}=\left(\frac{-2}{\omega^{-}(t)}\right)\frac{\sum_{m=0}^{N}\frac{dy_{m}(t)}{dt}}{\sum_{n=0}^{N}y_{n}(t)},$$

in which N is the order of approximation. For completeness, the derivatives of Eqs. ([Disp-formula Ch1.E26]) and ([Disp-formula Ch1.E29]) are, respectively, as follows:

31dy1(t)dt=c04ω^-(t)∫0tω^+(t′)dt′32dyn(t)dt=(c0+1)dyn-1(t)dt+c04ω^-(t)∫0tω^+(t′)yn-1(t′)dt′.

Results obtained using $y_{0}(t)=1$ , together with Eqs. ([Disp-formula Ch1.E26]), ([Disp-formula Ch1.E29]), and ([Disp-formula Ch1.E30]), will be called method 1 in the following discussion. The iterated form of the above expressions for $y_{n}(t)$ have similarities to the Fourier integrals obtained from average Hamiltonian theory by [Bibr bib1.bibx20].

The above choices of L[] and $y_{0}(t)$ are not unique. Different choices lead to different series approximations and, hence, to different qualitative and quantitative results. As a second example, Ω(t)=Ω is assumed to be fixed and only amplitude-modulated pulses ω(t) with x phase are considered (these assumptions can be relaxed as needed). Returning to Eq. ([Disp-formula Ch1.E8]), the homotopy is defined as follows:

33Ng(t)=d2g(t)dt2-dln⁡ω(t)dt+iΩdg(t)dt+14ω2(t)g(t)34Lg(t)=d2g(t)dt2-dln⁡ω(t)dtdg(t)dt+14ω2(t)g(t)35y0(t)=cos⁡12δ(t),36δ(t)=∫0tω(t′)dt′.

This choice of $y_{0}(t)$ satisfies the following:

37 $$\frac{d^{2}y_{0}(t)}{dt^{2}}-\frac{d\mathrm{ln}\left[\omega (t)\right]}{dt}\frac{dy_{0}(t)}{dt}+\frac{1}{4}\omega^{2}(t)y_{0}(t)=0,$$

and is the exact on-resonance solution for y(t) . Consequently, the first-order deformation equation leads to the following:

38 $$\begin{array}{rl} & \frac{d^{2}y_{1}(t)}{dt^{2}}-\frac{d\mathrm{ln}\left[\omega (t)\right]}{dt}\frac{dy_{1}(t)}{dt}+\frac{1}{4}\omega^{2}(t)y_{1}(t)\\ & \;\;\;\;\;=-ic_{0}\Omega \frac{dy_{0}(t)}{dt}. \end{array}$$

The solutions to the homogeneous equation (setting the right-hand side to 0) are $y^{\pm }(t)=e^{\pm i\delta (t)/2}$ . The method of variation of parameters then gives the inhomogeneous solution as follows:

39 $$y_{1}(t)=-ic_{0}\Omega \int_{0}^{t}\mathrm{sin}\left[\frac{\delta (t)}{2}-\frac{\delta (t^{\prime })}{2}\right]\mathrm{sin}\left[\frac{\delta (t^{\prime })}{2}\right]dt^{\prime }.$$

The n th-order deformation equation for n>1 is as follows:

40 $$\begin{array}{rl} \frac{d^{2}y_{n}(t)}{dt^{2}} & -\left[\frac{d\mathrm{ln}\left[\omega (t)\right]}{dt}+i\Omega \right]\frac{dy_{n}(t)}{dt}+\frac{1}{4}\omega^{2}(t)y_{n}(t)\\ & =(1+c_{0})\{\frac{d^{2}y_{n-1}(t)}{dt^{2}}-\frac{d\mathrm{ln}\left[\omega (t)\right]}{dt}\frac{dy_{n-1}(t)}{dt}\\ & \;\;\;\;\;\;\;+\frac{1}{4}\omega^{2}(t)y_{n-1}(t)\}-ic_{0}\Omega \frac{dy_{n-1}(t)}{dt}, \end{array}$$

with the following solution:

41 $$\begin{array}{rl} y_{n}(t)= & \left(1+c_{0})y_{n-1}(t\right)\\ & -ic_{0}\Omega \int_{0}^{t}\frac{2}{\omega (t^{\prime })}\mathrm{sin}\left[\frac{\delta (t)}{2}-\frac{\delta (t^{\prime })}{2}\right]\frac{dy_{n-1}(t^{\prime })}{dt^{\prime }}dt^{\prime }. \end{array}$$

Each $y_{n}(t)$ is proportional to $\Omega^{n}$ , and these results yield the following power series in Ω for y(t) :

42 $$y(t)=y_{0}(t)+\sum_{n=1}^{N}(2+c_{0})y_{n}(t),$$

which is substituted into Eq. ([Disp-formula Ch1.E30]) to obtain f(t) . Results using Eqs. ([Disp-formula Ch1.E39]), ([Disp-formula Ch1.E41]), and ([Disp-formula Ch1.E42]) will be called method 2 in the following discussion. For completeness, the derivatives of Eqs. ([Disp-formula Ch1.E39]) and ([Disp-formula Ch1.E41]) are as follows:

43dy1(t)dt=-ic0Ωω(t)2∫0tcos⁡δ(t)2-δ(t′)2sin⁡δ(t′)2dt′44dyn(t)dt=(1+c0)dyn-1(t)dt-ic0Ωω(t)2∫0t2ω(t′)×cos⁡δ(t)2-δ(t′)2dyn-1(t′)dt′dt′.

### 2.1 Methods

Numerical integration was performed using the trapezoid method implemented in Python 3.6. Pulse shapes were discretized in 1000 increments. Rectangular pulses were simulated using $\omega_{1}/(2\pi )=25\;000$ Hz and an on-resonance 90 ${}^{\circ }$ pulse length of 10.0 µs or $\omega_{1}/(2\pi )=250$ Hz and an on-resonance 90 ${}^{\circ }$ pulse length of 1 ms. EBURP-2 [Bibr bib1.bibx5] and Q5 [Bibr bib1.bibx4] pulses were simulated using a maximum $\omega_{1}/(2\pi )=9000$ Hz and 90 ${}^{\circ }$ pulse lengths of 455.2 and 504.9 µs , respectively. REBURP [Bibr bib1.bibx5] pulses were simulated using a maximum $\omega_{1}/(2\pi )=10\;000$ Hz and a 180 ${}^{\circ }$ pulse length of 626.5 µs . WURST-20 [Bibr bib1.bibx7] pulses were simulated using a maximum $\omega_{1}/(2\pi )=9512$ Hz, a frequency sweep of 50 000 Hz, and a pulse length of 440.0 µs .

Equation ([Disp-formula Ch1.E7]) can be recast as follows:

45 $$\begin{array}{rl} \alpha (\tau_{p})=\frac{i}{4}\int_{0}^{\tau_{p}} & dt\{\omega^{+}(t)f^{*}(t)-\omega^{-}(t)f(t)\}\\ & \times \{1+|f(t){|}^{2}\}/|f(t){|}^{2}, \end{array}$$

for numerical calculations. Alternatively, $\alpha (\tau_{p})$ can be obtained from the argument of $f(\tau_{p})$ calculated for the time-reversed pulse [Bibr bib1.bibx11]. The latter is more computationally demanding, but more numerically stable, and was used for the results presented herein.

### 2.2 Results and discussion

In the present applications, HAM converts the second-order Riccati differential equation, Eq. ([Disp-formula Ch1.E8]), which cannot be solved directly, into a series of second-order differential equations that have convenient solutions. The choice of $y_{0}(t)=1$ in method 1 leads to obtaining simple iterative solutions that can be calculated very efficiently. The form of $y_{0}(t)$ given in Eq. (36) could also be used in Eq. (24) to obtain an alternative expression for $y_{1}(t)$ to be substituted into Eqs. ([Disp-formula Ch1.E29]) and ([Disp-formula Ch1.E30]). The resulting first-order expressions for y(t) are usually more accurate than the first-order results obtained using $y_{0}(t)=1$ , but this advantage becomes less pronounced at higher orders of approximation and comes at increased computational cost. Thus, Eqs. ([Disp-formula Ch1.E26]), ([Disp-formula Ch1.E29]), and ([Disp-formula Ch1.E30]) are most suitable in practice.

A first example of the results of the above analysis is given for a rectangular 90 ${}^{\circ }$ pulse in Fig. [Fig Ch1.F1]. The integrals in Eqs. ([Disp-formula Ch1.E26]) and ([Disp-formula Ch1.E29]) can be performed analytically for a rectangular pulse with amplitude $\omega_{1}$ . For example, using Eq. ([Disp-formula Ch1.E26]) yields the following:

46 $$y_{1}(t)=\frac{c_{0}\omega_{1}^{2}}{4\Omega^{2}}\left(1-e^{i\Omega t}\right)+i\frac{c_{0}\omega_{1}^{2}t}{4\Omega };$$

however, analytic calculations of higher order $y_{n}(t)$ do not have advantages over numerical integration. As shown in Fig. [Fig Ch1.F1]a, b, the second- and third-order results obtained with method 1 and $c_{0}=-1$ are nearly indistinguishable from the exact result of Eq. ([Disp-formula Ch1.E3]) (using $\tau_{p}=\Delta \tau_{k}$ ) over the range of resonance offsets from 0 to $\Omega /\omega_{1}=15^{1/2}$ . The first-order result provides a highly accurate estimate of $\gamma (\tau_{p})$ but overestimates $\beta (\tau_{p})$ . The role of the convergence control parameter $c_{0}$ is illustrated in Fig. [Fig Ch1.F1]c, d. A value of $c_{0}=-0.925$ was chosen, using Eqs. ([Disp-formula Ch1.E46]) and ([Disp-formula Ch1.E30]), to scale the first-order result for $\beta (\tau_{p})$ to be equal to π/2 at Ω=0 . As shown, the resulting first-order result, using method 1, is now nearly exact at all resonance offsets. In the present application, adjusting the convergence control parameter provides accuracy equivalent to 1 or 2 additional higher orders of approximation. Remarkably, this same value of $c_{0}$ works well for a rectangular 180 ${}^{\circ }$ pulse (not shown) and 90 ${}^{\circ }$ EBURP-2, 90 ${}^{\circ }$ Q5, and 180 ${}^{\circ }$ REBURP and WURST inversion pulses (vide infra).

In contrast to the results of method 1, the power series for y(t) obtained, using method 2 with $c_{0}=-1$ , even to the third order in Ω , is accurate for $\beta (\tau_{p})$ only to slightly more than $\Omega /\omega_{1}=1$ . When $c_{0}=-0.925$ , the third-order power series has improved accuracy for resonance offsets up to nearly $\Omega /\omega_{1}=2$ . However, further increases in accuracy at larger resonance offsets require very large orders of approximation N in Eq. ([Disp-formula Ch1.E42]). For example, extending the accuracy of the power series for $\beta (\tau_{p})$ to offsets $\Omega /\omega_{1}=3.5$ requires N=50 . The differences between the results of method 1 and method 2 reflect the inevitable shortcomings of power series and perturbation approaches when the expansion parameter is not small.

**Figure 1 Ch1.F1:**
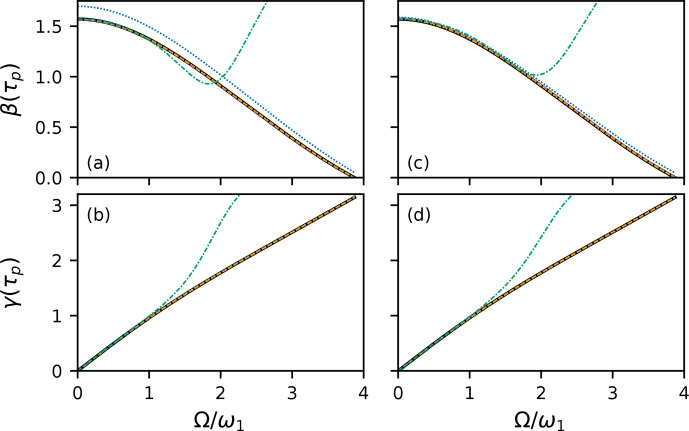
HAM approximations for on-resonance 90 ${}^{\circ }$ rectangular pulse with $\omega_{1}/(2\pi )=25\;000$ Hz. Exact calculation of Euler angles $\beta (\tau_{p})$ and $\gamma (\tau_{p})$ (black line). For a rectangular pulse, $\alpha (\tau_{p})=\gamma (\tau_{p})$ . First-order (blue dotted line), second-order (reddish-purple dashed line), and third-order (orange dash–dot–dotted line) HAM results using method 1. The third-order result is found using the power series of method 2 (green dash-dotted line). Results are shown for **(a, b)** $c_{0}=-1$ and **(c, d)** $c_{0}=-0.925$ . The exact, second-order HAM and third-order HAM curves for method 1 are virtually indistinguishable.

A more challenging example is given by the 90 ${}^{\circ }$ EBURP-2 pulse [Bibr bib1.bibx5]. In principle, the integrals in Eqs. ([Disp-formula Ch1.E26]) and ([Disp-formula Ch1.E29]) can be performed analytically because the pulse shape is expressed as a Fourier series (as are other pulses in the BURP [Bibr bib1.bibx5] and SNOB [Bibr bib1.bibx8] families). In practice, the number of terms that must be calculated becomes very large, and numerical integration is much more efficient. Calculations using method 1 are shown in Fig. [Fig Ch1.F2]. With $c_{0}=-1$ , the fifth-order approximation is extremely accurate compared with numerical calculations using Eqs. ([Disp-formula Ch1.E1])–([Disp-formula Ch1.E3]) (Fig. [Fig Ch1.F2]a–c). With $c_{0}=-0.925$ (Fig. [Fig Ch1.F2]d–f), even the small deviations observed for the fifth-order HAM approximation are eliminated, and the third-order result is accurate, except at the edge of the excitation band. In contrast, perturbation theory or power-series expansions (method 2) are extremely poor at reproducing $\beta (\tau_{p})$ , essentially failing as soon as Ω is non-zero (not shown). The accuracy of the method 1 approximations over the full range of resonance offsets shows that HAM, with an appropriate choice of linear operator and starting functions, can provide approximate solutions valid far beyond the range of perturbation theory.

**Figure 2 Ch1.F2:**
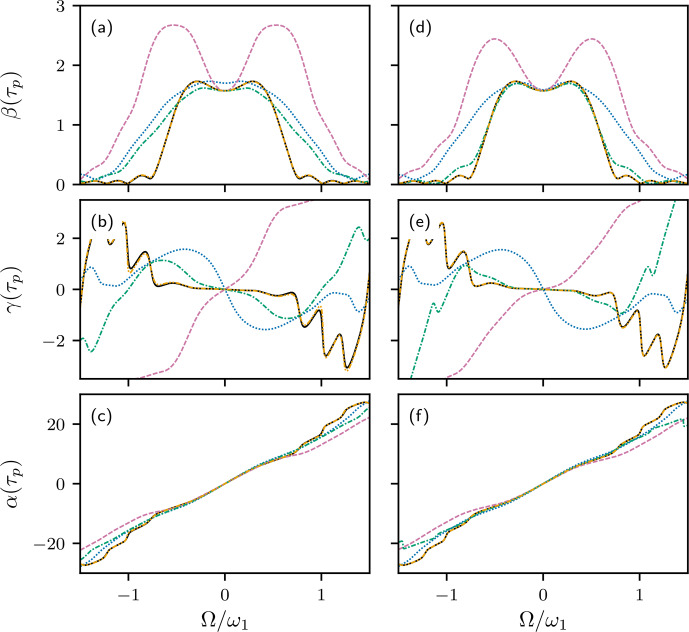
HAM approximations for 90 ${}^{\circ }$ EBURP-2 pulse. Numerical calculation of Euler angles $\alpha (\tau_{p})$ , $\beta (\tau_{p})$ , and $\gamma (\tau_{p})$ using Eqs. ([Disp-formula Ch1.E1])–([Disp-formula Ch1.E3]) (black line). First-order (blue dotted line), second-order (reddish-purple dashed line), third-order (green dash-dotted line), and fifth-order (orange dash–dot–dotted line) HAM results, using method 1. Results are shown for **(a, b, c)** $c_{0}=-1$ and **(d, e, f)** $c_{0}=-0.925$ . The numerical calculation and fifth-order HAM curves are nearly indistinguishable.

The Gaussian Q5 90 ${}^{\circ }$ pulse [Bibr bib1.bibx4] has a more complicated amplitude modulation profile than the EBURP-2 pulse and requires higher orders of approximation to obtain accurate results. Results obtained for method 1 with fifth- and seventh-order approximations are shown in Fig. [Fig Ch1.F3]. The seventh-order results are highly accurate for both $c_{0}=-1$ and $c_{0}=-0.925$ . The choice of $c_{0}=-0.925$ has a remarkable effect in terms of increasing the accuracy the fifth-order approximation to nearly that of the seventh-order result.

**Figure 3 Ch1.F3:**
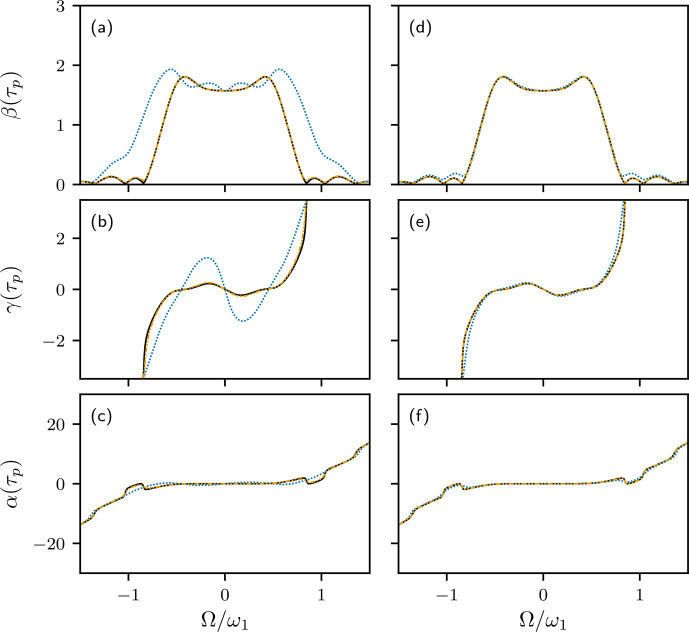
HAM approximations for 90 ${}^{\circ }$ Q5 pulse. Numerical calculation of Euler angles $\alpha (\tau_{p})$ , $\beta (\tau_{p})$ , and $\gamma (\tau_{p})$ using Eqs. ([Disp-formula Ch1.E1])–([Disp-formula Ch1.E3]) (black line). Fifth-order (blue dotted line) and seventh-order (orange dash–dot–dotted line) HAM results, using method 1. Results are shown for **(a, b, c)** $c_{0}=-1$ and **(d, e, f)** $c_{0}=-0.925$ . The numerical calculation and seventh-order HAM curves are nearly indistinguishable.

The application of HAM is not limited to 90 ${}^{\circ }$ pulses or to amplitude-modulated pulses. Figure [Fig Ch1.F4] shows the performance of method 1 for the 180 ${}^{\circ }$ REBURP [Bibr bib1.bibx5] and WURST-20 inversion [Bibr bib1.bibx7] pulses. As for the EBURP-2 pulse, the fifth-order approximation for the REBURP pulse is highly accurate for both $c_{0}=-1$ and $c_{0}=-0.925$ . The third-order approximation also is highly accurate when $c_{0}=-0.925$ . The WURST-20 pulse uses a linear frequency shift, generated by applying a quadratic phase shift during the pulse, and is an example of a phase-modulated or complex waveform. Again, the more complicated waveform requires higher order approximation, but 11th-order, with $c_{0}=-1$ , or ninth-order, with $c_{0}=-0.925$ , results are highly accurate.

**Figure 4 Ch1.F4:**
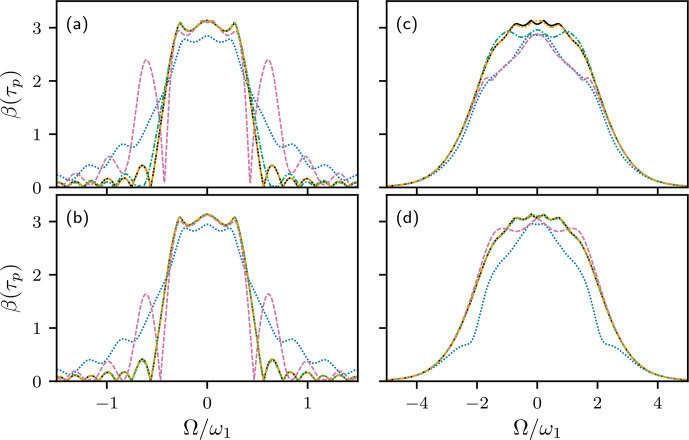
HAM approximations for **(a, b)** REBURP and **(c, d)** WURST-20 inversion pulses. Numerical calculation of Euler angle $\beta (\tau_{p})$ using Eqs. ([Disp-formula Ch1.E1])–([Disp-formula Ch1.E3]) (black line). **(a, b)** First-order (blue dotted line), second-order (reddish-purple dashed line), third-order (green dash-dotted line), and fifth-order (orange dash–dot–dotted line) HAM results, using method 1. **(c, d)** Fifth-order (blue dotted line), seventh-order (reddish-purple dashed line), ninth-order (green dash-dotted line), and 11th-order (orange dash–dot–dotted line) HAM results, using method 1. Results are shown for **(a, c)** $c_{0}=-1$ and **(b, d)** $c_{0}=-0.925$ . The numerical calculation and **(a, b)** fifth-order and **(c, d)** 11th-order HAM curves are nearly indistinguishable.

Method 2 yields a power series for y(t) . If $c_{0}=-1$ , the resulting series is identical to the power series expansion obtained from perturbation theory [Bibr bib1.bibx11], while $c_{0}=-0.925$ provides additional accuracy. However, as noted above, the power series requires very high orders N to obtain accuracy comparable to results from modest orders using method 1. Thus, method 1 is much more powerful for general calculations; however, the power series leads to a convenient expression for the near-resonance phase shift $\gamma (\tau_{p})$ . The first-order power series for y(t) , assuming $c_{0}=-1$ , yields the following:

f(t)=sin⁡δ(t)2+iΩ∫0tcos⁡δ(t)2-δ(t′)2sin⁡δ(t′)2dt′cos⁡δ(t)2-iΩ∫0tsin⁡δ(t′)2-δ(t′)2sin⁡δ(t′)2dt′47≈tan⁡δ(t)21+iΩsin⁡δ(t)∫0tsin⁡δ(t′)dt′,

in which the second equality is the expansion to first order in Ω , and the resulting trigonometric functions have been simplified. This result is identical to the previously reported result from the first-order perturbation theory [Bibr bib1.bibx11]. The argument of the first-order approximation of f(t) is a good estimate of the phase $\gamma (\tau_{p})$ of the transverse magnetization following the pulse. As noted above, the phase α(t) is obtained by repeating the calculation with the time-reversed pulse. Therefore, as concluded from the perturbation theory, an amplitude-modulated shaped pulse acts as an ideal rotation of the angle $\beta (\tau_{p})$ preceded and followed by time delays $\tau_{\alpha }$ and $\tau_{\gamma }$ over the frequency range for which the first-order approximation holds [Bibr bib1.bibx9], as in the following:

48τα=1sin⁡δ(τp)∫0τpsin⁡δ(τp-t′)dt′49τγ=1sin⁡δ(τp)∫0τpsin⁡δ(t′)dt′.

For a 90 ${}^{\circ }$ pulse, the above equations can be written compactly as follows:

50 $$\tau_{\alpha }+i\tau_{\gamma }=\int_{0}^{\tau_{p}}e^{i\delta (t^{\prime })}dt^{\prime }.$$

The ratios $\tau_{\alpha }/\tau_{p}$ and $\tau_{\gamma }/\tau_{p}$ are the average projections of a unit vector onto the z axis and -y axis, respectively, over the duration of the pulse (for a vector is oriented along the z axis at time 0 ).

The above explications have focused on solutions to the transformed Riccati equation in Eq. ([Disp-formula Ch1.E8]). However, HAM also could be applied directly to the original Riccati equation of Eq. ([Disp-formula Ch1.E5]). For example, by analogy to method 2, choosing the following:

51N[g(t)]=dg(t)dt-12ω(t)g2(t)-iΩg(t)-12ω(t)52L[g(t)]=dg(t)dt-iΩg(t)53f0(t)=tan⁡δ(t)2,

in which $f_{0}(t)$ is the exact solution for Ω=0 , yields the following series solution:

54 $$f(t)=\mathrm{tan}\left[\frac{\delta (t)}{2}\right]+\sum_{n=1}^{N}f_{n}(t).$$

The result obtained from the first-order deformation equation is as follows:

df1(t)dt-iΩf1(t)=-ic0Ωf0(t)55f1(t)=-ic0ΩeiΩt∫0te-iΩttan⁡δ(t′)2dt′.

However, additional terms in the series lack the simple iterative structure shown in Eqs. ([Disp-formula Ch1.E29]) and ([Disp-formula Ch1.E41]) because of the increasing complexity of the higher derivatives of $\Phi^{2}(t;q)$ that must be calculated for the n th order deformation equation. For example, the differential equations for the next two terms in the series for f(t) become the following:

56 $$\begin{array}{rl} \frac{df_{2}(t)}{dt} & -i\Omega f_{2}(t)\\ & =c_{0}\{\frac{df_{1}(t)}{dt}-i\Omega f_{1}(t)-\omega (t)f_{0}(t)f_{1}(t)\}. \end{array}$$

57 $$\begin{array}{rl} \frac{df_{3}(t)}{dt} & -i\Omega f_{3}(t)\\ & =c_{0}\{\frac{df_{2}(t)}{dt}-i\Omega f_{2}(t)-2\omega (t)f_{0}(t)f_{2}(t)-\omega (t)f_{1}^{2}(t)\}. \end{array}$$

In addition, results obtained using Eq. ([Disp-formula Ch1.E30]) to obtain f(t) from y(t) generally are more accurate than results obtained by direct calculation of f(t) at the same order of approximation. Thus, in this particular application, use of HAM with the transformed Riccati equation, Eq. ([Disp-formula Ch1.E8]), yields more convenient expressions. Nonetheless, this example demonstrates the particular power of HAM in directly converting the solution of a nonlinear differential equation into a series of linear first-order differential equations, which always can be solved by integration [Bibr bib1.bibx13].

For many applications, the Euler angles for a shaped pulse are easily obtained from Eqs. ([Disp-formula Ch1.E1])–([Disp-formula Ch1.E3]). However, calculations for method 1 using Eqs. ([Disp-formula Ch1.E26]), ([Disp-formula Ch1.E29]), and ([Disp-formula Ch1.E30]) are extremely efficient. In Python 3.6, the seventh-order HAM approximation for the Q5 pulse is approximately 20 times faster than direct calculations using Eqs. ([Disp-formula Ch1.E1])–([Disp-formula Ch1.E3]). Thus, these approximations may be particularly useful for the computational design of radiofrequency pulses in which many iterations of a search or optimization routine are necessary [Bibr bib1.bibx6].

The Euler angle representation is particularly convenient because, once calculated, the Euler angles can be used to determine the outcome of a shaped pulse applied to arbitrary initial magnetization. The Riccati equation can be extended to incorporate radiation damping, but not relaxation, as discussed by [Bibr bib1.bibx15]. However, the Euler angles can serve to generate the initial approximations for a second application of HAM to obtain approximate solutions to the Bloch equations for particular initial conditions, including relaxation. In the following, Ω(t)=Ω is assumed to be fixed, and only amplitude-modulated pulses ω(t) with x phase are considered (these assumptions can be relaxed as needed). The Bloch equations for a pulse applied with x phase can be written in the following form:

58 $$\frac{d}{dt}\hat{M}(t)=-\Gamma (t)\hat{M}(t)+\left[\begin{array}{c} 0\\ 0\\ e^{R_{2}t}R_{1}M_{0} \end{array}\right],$$

59 $$\hat{M}(t)=\left[\begin{array}{c} {\hat{M}}_{x}(t)\\ {\hat{M}}_{y}(t)\\ {\hat{M}}_{z}(t) \end{array}\right],$$

60 $$\Gamma (t)=\left[\begin{array}{ccc} 0 & \Omega & 0\\ -\Omega & 0 & \omega_{x}(t)\\ 0 & -\omega_{x}(t) & -(R_{2}-R_{1}) \end{array}\right],$$

in which $M_{k}(t)=e^{-R_{2}t}{\hat{M}}_{k}(t)$ are the Cartesian components of the magnetization, and $M_{0}$ is the equilibrium magnetization. The use of transformed variables $\hat{M}(t)$ rather than M(t) simplifies the following discussion. The linear operator is chosen as L[g(t)]=dg(t)/dt , in which $g(t)=[g_{x}(t),g_{y}(t),g_{z}(t){]}^{T}$ is an arbitrary vector function. The nonlinear operator is as follows:

61 $$N[g(t)]=\frac{dg(t)}{dt}+\Gamma (t)g(t)-\left[\begin{array}{c} 0\\ 0\\ e^{R_{2}t}R_{1}M_{0} \end{array}\right].$$

The initial zeroth-order approximations for HAM are ${\hat{M}}_{0}(t)=M_{0}(t)$ and are given by the solutions to the Bloch equations in the absence of exchange for initial equilibrium magnetization $[0,0,M_{0}{]}^{T}$ . The initial approximations are calculated by using the Euler angles determined from method 1 described above. Thus, in the following:

62 $$\frac{d}{dt}{\hat{M}}_{0}(t)+\Gamma (t){\hat{M}}_{0}=0,$$

and the system of first-order deformation equations yield the following:

63 $$\frac{d}{dt}{\hat{M}}_{1}(t)=-c_{0}\left[\begin{array}{c} 0\\ 0\\ (R_{2}-R_{1}){\hat{M}}_{z0}(t)+e^{R_{2}t}R_{1}M_{0} \end{array}\right].$$

The solution to this equation yields ${\hat{M}}_{x1}(t)={\hat{M}}_{y1}(t)=0$ , and ${\hat{M}}_{z1}(t)$ is given by the following:

64 $$\begin{array}{rl} & {\hat{M}}_{z1}(t)\\ & =-c_{0}\left[(R_{2}-R_{1})\int_{0}^{t}{\hat{M}}_{z0}(t^{\prime })dt^{\prime }+\frac{R_{1}}{R_{2}}(e^{R_{2}t}-1)M_{0}\right]\\ & =-c_{0}\left[(R_{2}-R_{1})t<{\hat{M}}_{z0}(t)>+\frac{R_{1}}{R_{2}}(e^{R_{2}t}-1)M_{0}\right]. \end{array}$$

The first-order approximation of magnetization during the pulse is given by the following:

65 $$M(t)=e^{-R_{2}t}\left[\begin{array}{c} M_{x0}(t)\\ M_{y0}(t)\\ M_{z0}(t)+{\hat{M}}_{z1}(t) \end{array}\right].$$

At this level of approximation, relaxation of transverse magnetization depends simply on $R_{2}$ , while relaxation of $M_{z}(t)$ depends on the average z magnetization during the pulse (calculated in the absence of relaxation). For macromolecules, $R_{2}\gg R_{1}$ typically, and the term proportional to $R_{1}/R_{2}$ is small.

The n th-order deformation equation leads to the following expression for n>1 :

66 $${\hat{M}}_{n}(t)=(1+c_{0}){\hat{M}}_{\left(n-1\right)}(t)-c_{0}\int_{0}^{t}\Gamma (t^{\prime }){\hat{M}}_{\left(n-1\right)}(t^{\prime })dt^{\prime }.$$

If $c_{0}=-1$ , the above recursive expressions can be written compactly as follows:

67 $$\begin{array}{rl} {\hat{M}}_{n}(t)= & \;(-1{)}^{n-1}\int_{0}^{t}\Gamma (t_{n-1})dt_{n-1}\int_{0}^{t_{n-1}}\Gamma (t_{n-2})dt_{n-2}\ldots \\ & \;\;\;\times \int_{0}^{t_{2}}\Gamma (t_{1}){\hat{M}}_{1}(t_{1})dt_{1}. \end{array}$$

For a rectangular pulse applied to equilibrium magnetization (with magnitude set to unity for convenience), the initial approximations are as follows:

68 $$M_{0}(t)=\left[\begin{array}{c} {}[1-\mathrm{cos}(\omega_{e}t)]\mathrm{cos}\theta \mathrm{sin}\theta \\ -\mathrm{sin}(\omega_{e}t)\mathrm{sin}\theta \\ \mathrm{cos}(\omega_{e}t){\mathrm{sin}}^{2}\theta +{\mathrm{cos}}^{2}\theta \end{array}\right],$$

69 $$\begin{array}{rl} {\hat{M}}_{z1}(t)=-c_{0} & (R_{2}-R_{1})\left[\frac{1}{\omega_{e}}\mathrm{sin}(\omega_{e}t){\mathrm{sin}}^{2}\theta +t{\mathrm{cos}}^{2}\theta \right]\\ & -c_{0}\frac{R_{1}}{R_{2}}(e^{R_{2}t}-1). \end{array}$$

In this case, Γ(t)=Γ and the series of approximations given in Eq. ([Disp-formula Ch1.E67]) can be summed to give the following:

70 $$\begin{array}{rl} M(t)=e^{-R_{2}t} & \{M_{0}(t)+\int_{0}^{t}e^{\Gamma (t-t^{\prime })}\\ & \times \left[\begin{array}{c} 0\\ 0\\ (R_{2}-R_{1})M_{z0}(t^{\prime })+e^{R_{2}t^{\prime }}R_{1}M_{0} \end{array}\right]dt^{\prime }\}, \end{array}$$

and this yields identical results as a direct integration of the Bloch equations. Equations ([Disp-formula Ch1.E65]), ([Disp-formula Ch1.E67]), and ([Disp-formula Ch1.E70]) explicitly show the effect of relaxation as a perturbation of the evolution of magnetization in the absence of relaxation.

Figure [Fig Ch1.F5] shows the magnetization components for rectangular $90{}^{\circ }$ , $180{}^{\circ }$ , $270{}^{\circ }$ , and $360{}^{\circ }$ nominal on-resonance pulses in the absence and presence of relaxation. Calculations were performed in the absence of relaxation using Eq. ([Disp-formula Ch1.E68]) and in the presence of relaxation using the HAM approximations, i.e., Eqs. ([Disp-formula Ch1.E69]) and ([Disp-formula Ch1.E70]). The first-order HAM approximation is surprisingly accurate for moderate values of $R_{2}$ , except for cases in which $<{\hat{M}}_{z0}(t)>=0$ , such as the on-resonance $360{}^{\circ }$ pulse. The above expressions display the fundamental dependence of relaxation during a pulse applied to equilibrium magnetization on the time-average z magnetization.

**Figure 5 Ch1.F5:**
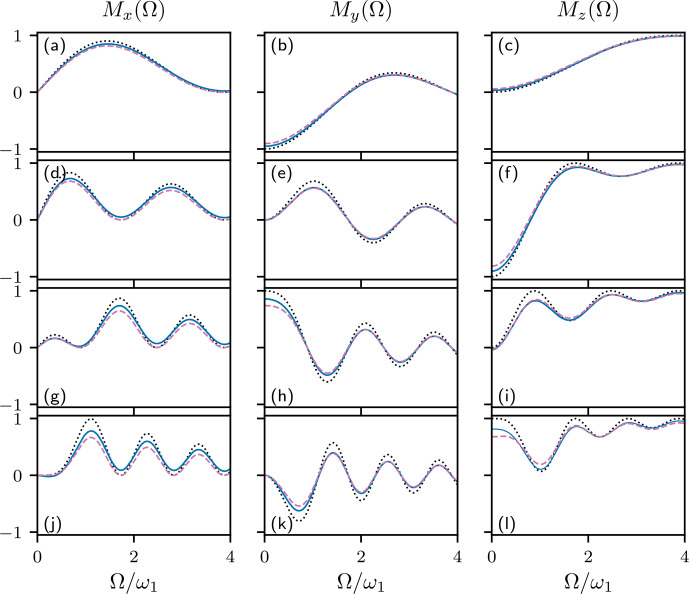
HAM approximations for rectangular **(a, b, c)** 90 ${}^{\circ }$ , **(d, e, f)** 180 ${}^{\circ }$ , **(g, h, i)** 270 ${}^{\circ }$ , and **(j, k, l)** 360 ${}^{\circ }$ pulses applied to initial z magnetization. Values of **(a, d, g, j)** $M_{x}(\Omega )$ , **(b, e, h, k)** $M_{y}(\Omega )$ , and **(c, f, i, l)** $M_{z}(\Omega )$ are shown as functions of resonance offset Ω . Magnetization components in the absence of exchange, using Eq. ([Disp-formula Ch1.E68]) (black dotted line), first-order HAM approximation of the Bloch equations using Eq. ([Disp-formula Ch1.E69]) (reddish-purple dashed line), and exact HAM solution of the Bloch equations using Eq. ([Disp-formula Ch1.E70]) (blue solid line). Calculations used $\omega_{1}/(2\pi )=250$ Hz, $R_{1}=2\;s^{-1}$ , $R_{2}=100\;s^{-1}$ , and $c_{0}=-1$ .

## 3 Conclusion

Fast, accurate methods for solving differential equations have widespread application in NMR spectroscopy. The present work has illustrated the homotopy analysis method [Bibr bib1.bibx13] for approximating solutions for differential equations by application to the Riccati differential equation for the Euler angle representation of radiofrequency pulse shapes and to solutions of the Bloch equations incorporating relaxation. The freedom to select the linear operator, lowest-order approximate solution, convergence control parameter, and auxiliary function is powerful in obtaining series solutions that are highly accurate for low orders of approximation and efficient to calculate or that provide qualitatively convenient series allowing physical insight. It can be expected that homotopy analysis method will find other applications in NMR spectroscopy.

## Supplement

10.5194/mr-2-175-2021-supplement The supplement related to this article is available online at: https://doi.org/10.5194/mr-2-175-2021-supplement.

## Data Availability

RMarkdown and bibtex files are provided in the Supplement. The RMarkdown file contains the Python code for all calculations described in the paper.
