# Supplementary material for: Approximate representations of shaped pulses using the homotopy analysis method
Source: Magn Reson (Gott). 2021 Apr 16;2(1):175–86. doi: 10.5194/mr-2-175-2021 (PMC8372782; doi:10.5194/mr-2-175-2021)
Supplement: The supplement related to this article is available online at: https://doi.org/10.5194/mr-2-175-2021-supplement. [file mr-2-175-supplement.zip › mr-2-175-2021-supplement-title-page.pdf]

Supplement of Magn. Reson., 2, 175–186, 2021
https://doi.org/10.5194/mr-2-175-2021-supplement
© Author(s) 2021. CC BY 4.0 License.

Open Access
MAGNETIC RESONANCE

Supplement of

Approximate representations of shaped pulses using the homotopy analysis method

Timothy Crawley and Arthur G. Palmer III

Correspondence to: Arthur G. Palmer III (agp6@columbia.edu)

- mr-2-175-2021-supplement-title-page.pdf
- Crawley_HAM_Supplement
  - .DS_Store
  - Crawley_HAM_ver4.2.Rmd
  - HAM.bib
- __MACOSX
  - Crawley_HAM_Supplement
    - ._.DS_Store
    - ._Crawley_HAM_ver4.2.Rmd
    - ._HAM.bib

The copyright of individual parts of the supplement might differ from the article licence.
